# Clinical indicators of *dry eye severity* nursing outcome in intensive care unit*

**DOI:** 10.1590/1518-8345.2983.3201

**Published:** 2019-10-28

**Authors:** Raffaela Patrícia da Silva Soares, Ana Paula Nunes de Lima Fernandes, Fabiane Rocha Botarelli, Jéssica Naiara de Medeiros Araújo, Jéssica de Araújo Olímpio, Allyne Fortes Vitor

**Affiliations:** 1 Universidade Federal do Rio Grande do Norte, Departamento de Enfermagem, Natal, RN, Brazil.; 2 Secretaria Municipal de Saúde, Unidade de Pronto Atendimento Dr. Luiz Lindbergh Farias, João Pessoa, PB, Brazil.; 3 Scholarship holder at the Coordenação de Aperfeiçoamento de Pessoal de Nível Superior (CAPES), Brazil.; 4 Universidade Federal do Rio Grande do Norte, Faculdade de Ciências da Saúde do Trairi, Santa Cruz, RN, Brazil.; 5 Hospital do Coração de Natal, Natal, RN, Brazil.

**Keywords:** Nursing, Intensive Care Units, Dry Eye Syndromes, Eye Health, Nursing Assessment, Nursing Process, Enfermagem, Unidades de Terapia Intensiva, Síndromes do Olho Seco, Saúde Ocular, Avaliação em Enfermagem, Processos de Enfermagem, Enfermería, Unidades de Terapia Intensiva, Síndromes del Ojo Seco, Salud Ocular, Evaluación en Enfermería, Procesos de Enfermería

## Abstract

**Objective:**

to verify the extent of impairment of the clinical indicators of the nursing outcome Dry Eye Severity in patients admitted to the Intensive Care Unit.

**Method:**

cross-sectional, descriptive study developed with 206 patients. Based on the result listed, six indicators of the Classification of Nursing Results were evaluated with a questionnaire containing clinical variables and the Likert scale of the Classification of Nursing Results with constructed definitions, which varies from more impaired to non-impaired. The data were analyzed using descriptive and inferential statistics.

**Results:**

the decrease in lacrimal production and the presence of redness in the conjunctiva were more impaired. The other indicators were more frequent for the absence of impairment: incomplete eyelid closure 81% (167), excessive tearing 95.1%(196), excessive mucous secretion 78.7% (162) and decreased blinking mechanism 50.5% (104). The clinical characteristics of hospitalization for neurological disorders, invasive mechanical ventilation, chemosis, use of sedatives, vasoconstrictors, benzodiazepines, antibiotics and corticosteroids interfered in the impairment of the dry eye severity.

**Conclusion:**

the result indicators show that the clinical characteristics of patients in the intensive care unit interfere in the impairment and in the dry eyes severity. According to these results, the importance of assistance directed to the prevention of eye diseases is emphasized.

## Abstract (pt)

**Objetivo:**

verificar o grau de comprometimento dos indicadores clínicos do Resultado de Enfermagem *Gravidade do Olho Seco* em pacientes internados em unidades de terapia intensiva.

**Método:**

estudo transversal e descritivo desenvolvido com 206 pacientes. Do resultado elencado, seis indicadores da classificação dos Resultados de Enfermagem foram avaliados com questionário contendo variáveis clínicas e com a escala de Likert com definições construídas, que varia de mais comprometido a sem comprometimento. Os dados foram analisados mediante estatística descritiva e inferencial.

**Resultados:**

a diminuição da produção lacrimal e a presença de vermelhidão na conjuntiva estiveram associadas com maior comprometimento. Os demais indicadores foram mais frequentes para a ausência de comprometimento: fechamento palpebral incompleto 81% (167), lacrimejamento excessivo 95,1% (196), excesso de secreção mucosa 78,7% (162) e mecanismo de piscar diminuído 50,5% (104). As características clínicas internação por distúrbios neurológicos, ventilação mecânica invasiva, quemose, uso de sedativos, vasoconstrictores, benzodiazepínicos, antibióticos e corticoides interferiram no comprometimento na gravidade do olho seco.

**Conclusão:**

os indicadores do resultado mostram que as características clínicas dos pacientes da unidade de terapia intensiva interferem no comprometimento e gravidade do olho seco. A partir das relações, ressalta-se a importância da assistência direcionada à prevenção de agravos oculares.

## Abstract (es)

**Objetivo:**

verificar el grado de deterioro de los indicadores clínicos del resultado de enfermería *Gravedad del ojo seco* en pacientes ingresados en la Unidad de Cuidados Intensivos.

**Método:**

estudio descriptivo transversal desarrollado con 206 pacientes. A partir del resultado listado, se evaluaron seis indicadores de la Clasificación de Resultados de Enfermería con un cuestionario que contenía variables clínicas y la escala Likert de la Clasificación de Resultados de Enfermería con definiciones construidas, que varía desde más deteriorada hasta no deteriorada. Los datos se analizaron mediante estadísticas descriptivas e inferenciales.

**Resultados:**

La disminución de la producción lagrimal y el enrojecimiento en la conjuntiva mostraron un mayor deterioro. Los otros indicadores fueron más frecuentes en ausencia de deterioro: cierre incompleto de párpados 81% (167), lagrimeo excesivo 95.1% (196), secreción mucosa excesiva 78.7% (162), y disminución del mecanismo de parpadeo 50.5% (104). Las características clínicas de la hospitalización por trastornos neurológicos, ventilación mecánica invasiva, quemosis, uso de sedantes, vasoconstrictores, benzodiacepinas, antibióticos y corticosteroides interfieren en el deterioro en la gravedad del ojo seco.

**Conclusión:**

Los indicadores de resultados muestran que las características clínicas de los pacientes de la unidad de cuidados intensivos interfieren en el deterioro y en la gravedad del ojo seco. A partir de estas relaciones, se enfatiza la importancia de la asistencia dirigida a la prevención de enfermedades oculares.

## Introduction

Dry eye syndrome is defined as a multifactorial disease of the ocular surface characterized by loss of tear film homeostasis, with ocular symptoms, in which the instability and hyperosmolarity of the tear film, inflammation and damage of the ocular surface and neurosensory abnormalities have etiological significance^(^ 1 ^)^.

The most common risk factors for Dry Eye occurrence include: age over 60 years; female gender, especially those women receiving estrogen replacement therapy; wearing contact lenses; low humidity environment; systemic drugs (antihypertensives, diuretics, sedatives, neuromuscular blockers, benzodiazepines, anti-inflammatories, antihistamines, antibiotics, corticosteroids and vasoactive drugs) and autoimmune disorders (diabetes mellitus, sjögren syndrome, thyroid disease)^(^ 1 ^-^ 2 ^)^. The diagnosis of ocular surface disease is based on the signs, symptoms and clinical history of the patient associated with some quantitative and qualitative tests^(^ 2 ^)^.

Dry Eye Workshop II (DEWS II) indicates that this is a global problem, affecting more than 30 million people in the United States, and is one of the most frequent causes of demand for specialized eye care. In Asia and Europe, the prevalence of the syndrome, with and without symptoms, varies widely from 5% to 50% and its prevalence based only on signs is even more variable, reaching up to 75% in some populations^(^ 1 ^)^.

In Brazil, in the Intensive Care Unit (ICU), its occurrence becomes a concern. A previous cohort study indicates the presence of a global incidence of 53% of the outcome in patients hospitalized during the evaluated period^(^ 3 ^)^.

In these units, this problem becomes relevant, since patients admitted in this context are often in very critical health situations that increase the risk of developing ocular alterations. In addition, they are continuously exposed to environmental factors such as low temperature and humidity, which contribute to the occurrence of eye dryness and consequently Dry Eye^(^ 3 ^)^.

In this perspective, the adoption of a preventive approach and the implementation of eye care is very important for ICU patients; however, there is a prioritization of more urgent and complex care. The nursing team, responsible for comprehensive and systematized care, provides more time of assistance to these patients, by identifying risk factors and adopting preventive measures^(^ 4 ^)^.

This study is regulated by the *Sistematização da Assistência de Enfermagem* - SAE (Systematization of Nursing Assistance), operated by the Nursing Process (NP) in five stages (historical, diagnostics, planning, implementation and evaluation of nursing) and guided by the NANDA International, Inc.(NANDA-I) classification systems^(^ 5 ^)^, Nursing Interventions Classification (NIC)^(^ 6 ^)^ and Nursing Outcome Classification (NOC)^(^ 7 ^)^. It is based on the Nursing Diagnosis (ND) (00219) Risk of dry eyes of taxonomy of NANDA-I specified as “Susceptibility to eye discomfort or damage to the cornea and conjunctiva due to reduced amount or quality of tears to hydrate the eye, which can compromise health.”^(^ 5 ^)^; and, especially, in the Nursing Outcome (NO) (2110) Dry Eye Severity of the NOC taxonomy, defined as “severity of signs and symptoms of insufficient tears”^(^ 7 ^-^ 8 ^)^.

The Nursing Outcome Dry Eye Severity offers to the nurses a complete set of data to guide decisions, establish goals and classify uniformly the assessment of the health status of patients and direct the provision of care^(^ 8 ^)^. The Likert-type scale scores correspond to each indicator; the measurement of the result establishes a baseline score and classify the result obtained after the intervention. This scale varies from a more impaired value (1) to a non-impaired one (5)^(^ 7 ^)^.

The NOC presents advantages such as reduced documentation time, better patient satisfaction and results, greater uniformity in the evaluation of the professional nurse and improved quality of care^(^ 9 ^-^ 10 ^)^. This study is justified by the importance of developing a clinical evaluation focused on the condition of eye health and prevention of adverse events to the patient, using a taxonomy composed of a set of indicators to direct the care and determine interventions that provide positive results. Therefore, it is based on the following guiding question: What is the degree of ocular impairment of patients in the ICU?

In this perspective, the objective is to verify the extent of impairment of the clinical indicators of the nursing outcome Dry Eye Severity in patients admitted to the Intensive Care Unit.

## Method

This cross-sectional, descriptive, quantitative study was part of a larger study developed during the period from January to July 2016, in a university hospital located in Natal/RN. Strengthening the Reporting of Observational Studies in Epidemiology (STROBE) was used as an instrument to verify the items necessary for the methodological quality of the study^(^ 11 ^)^.

The target population consisted of adult patients admitted to the Intensive Care Unit. The sampling was done by convenience and resulted from the consecutive selection of participants as they were admitted to the ICU and met the inclusion criteria. The sample was established by applying the calculation to finite populations, n = Z^2^_a_.p.q.N/[Z_a_^2^.p.q+(N-1).e^2^], obtaining 206 patients.

The inclusion criteria adopted were minimum time of 24 hours in the ICU; age equal to or greater than 18 years. Patients submitted to any ophthalmologic intervention or under topical therapeutic eye treatment during data collection were excluded.

An instrument composed of variables related to sociodemographic data was applied for the operationalization of the data collection stage. The variable was clinical characteristics (type of admission, reason for admission, associated comorbidities, use of sedatives, invasive mechanical ventilation, chemosis, Ramsay scale, Glasgow coma scale and cranial nerve pairs III, IV and VI, use of medications, laboratory tests); risk factors of ND Risk of Dry Eyes of taxonomy NANDA-I^(^ 5 ^)^ and clinical indicators of the NO Dry Eye Severity of taxonomy NOC^(^ 7 ^)^. Data were obtained by primary source (patient and researcher’s observation) and by secondary source (analysis of medical records).

To measure these variables, the following devices were standardized: thermometer Incoterm^®^ for measuring air humidity and ambient temperature; medical pen-type flashlight for ocular evaluation; monitor Dixtal^®^ sector-specific for verification of vital signs and the Schirmer I test of the Ophtalmos^®^ brand for the measurement of tear volume. The Schirmer I test consists of placing small strips of sterile filter paper under the eyelid in the lower fornix near the lateral corner, away from the cornea. The eyelid is closed for five minutes and the wet portion of the strip is measured in millimeters. Values of less than 10 mm/5 min indicate low volumetry of the tear film^(^ 12 ^)^. The variables chosen as clinical characteristics were selected by the researchers based on the research that presented relationships between the elements of the phenomenon in their state-of-the-art^(^ 1 ^-^ 3 ^,^ 8 ^)^.

In order to avoid measurement biases, in the period prior to data collection, an eight hours training was carried out to two distinct teams: evaluators and diagnosticians. The first team of nurses and nursing scholars members of the *Núcleo de Ensino e Pesquisa em Enfermagem Clínica* - NEPEC (Center for Teaching and Research in Clinical Nursing) of the Universidade Federal do Rio Grande do Norte (UFRN) designated for data collection was trained based on relevant issues involving the theme, composition of the instrument variables and operationalization of the Schirmer I test. Then, the team was submitted to theoretical and practical evaluations and considered able after obtaining an average above 7.0.

The second team composed of nurses who diagnose, also members of the NEPEC and selected from attributes of knowledge and clinical and scientific experience involving the context of this research, had a specific training, including weekly discussions about Dry Eye and the ND Risk of Dry Eyes, with the purpose of providing accuracy in diagnostic inference. The recognition of patterns, a process of reasoning including investigation, collection, interpretation, grouping and validation of data was used as a guide for the study from the results of the pilot study, applied to 30 patients, in order to test and adapt the collection instrument. The diagnosticians were considered trained when the degree of agreement measured by the Kappa coefficient showed values between 0.81 and 1.00 (almost perfect).

After data collection made by the evaluators, the team of diagnosticians received a spreadsheet with the patients’ data for diagnostic inference of the presence of Risk of Dry Eyes, using the identification of the ND elements applicable to the population under study. These elements are: risk factors (air conditioning, smoking, excess wind and low humidity); populations at risk (aging, female gender and history of allergy) and associated conditions (autoimmune disease, neurological injury with loss of sensory or motor reflex and treatment regimen) or Dry Eye, from the Schirmer Test criteria < 10 mm associated with the presence of hyperemia and/or ocular secretion, according to adaptation to DEWS II recommendations. The elements for the inference of ND and Dry Eye were previously evaluated by observation of the researcher, records of medical records and use of specific materials already mentioned above.

After this intervention, the degree of impairment was evaluated by the NO Dry Eye Severity using an instrument with constitutive, operational definitions and operational magnitudes^(^ 8 ^)^ in order to achieve greater accuracy in the evaluation and reduce the subjectivity of clinical judgment. It was used after prior authorization by the author of the instrument, as a tool for the analysis of the clinical indicators of choice for the study, as shown in Figure 1.

**Figure 1:** Operational magnitudes of the indicators of the Nursing Result Dry Eye Severity^( 8 )^

	Decrease in tear production	Incomplete eyelid closing	Conjunctiva redness	Excessive tearing	Excessive mucous secretion	Decreased blinking mechanism
**1**	< or = 2 mm	Completely exposed cornea	Numerous vessels all over the ocular surface	Continuous tears	Purulent	< or = 2 times/min
**2**	< or = 5 mm	½ of the exposed cornea	Numerous vessels in the conjunctiva and at the beginning of the cornea	Tears running down the face	Mucopurulent	3 times/min
**3**	< or = 10 mm	1/3 lower of the exposed cornea	Expanded vessels in the conjunctiva and perilimbal	Occasional Coryza	Mucous plaques	4 times/min
**4**	or = 15 mm	Exposed conjunctive	Superficial hyperemia in the conjunctiva	Slightly wet eyes	Mucoid filaments	5 times/min
**5**	> 15 mm	Absent	Absent	Absent	Absent	>5 times/min

Although the NOC presents 14 indicators for the NO Dry Eye Severity. Only six indicators related to signs were listed to be measured, because the clinical conditions of most patients in the ICU hinder them talk about their symptoms, making it impossible to measure subjective indicators, such as: burning sensation in the eyes, sensation of eye itching, sandy sensation, foreign body sensation, eye pain, blurred vision, sensitivity to light and eye fatigue.

Therefore, considering the objective data to standardize the sample, the degree of agreement measured by the Kappa coefficient after the evaluation performed by the team of diagnosticians regarding the presence of ND Risk of Dry Eyes or Dry Eye, by means of clinical signs, lacrimal volumetry test and risk factors was 0.941 for right eyes inference and 0.961 for left eyes (almost perfect agreement). Then, the evaluation of the NO Dry Eye Severity was made, with the appropriate Likert scale score for each selected indicator.

The instrument of the NO Dry Eye Severity with the definitions constructed in a previous study^(^ 8 ^)^ was submitted to content validation in two stages, the first by specialists and the second with a consensus group.

The first step occurred with a content validation made by experts. These judges were selected according to their academic skills, development of studies and works in the area of eye health and nursing process and by their professional experience in the assistance and teaching of these themes.

A sample of 22 specialists evaluated the instrument in terms of psychometric criteria: behavior, objectivity, simplicity, relevance and precision. The binomial test was applied to analyze the agreement between the specialists, and the items presenting values from 85% of agreement were evaluated as adequate.

In order to synthesize the suggestions given by the judges and to refine the items that presented lower agreement in some psychometric criterion, the second stage of content validation was performed with a consensus group. This step included nine nurses, selected according to their qualifications and practical experience or teaching about the phenomenon studied. The consensus occurred after two meetings to discuss the theoretical aspect of the items.

For the statistical analysis, all data were organized, grouped, exported to a database and then analyzed by the software Statistical Package for Social Science (SPSS) version 22.0 for testing. Frequencies, measurements of the distribution center and its variabilities were used for descriptive analysis. Data normality was verified using the Shapiro-wilk test.

After a descriptive analysis of the distribution of scores, an inferential analysis was performed between the outcome indicators under study with the clinical characteristics and medications used by patients in the ICU, which considered the indicators that showed a relationship with the variables listed by pertinence.

To perform this crossing, the NOC indicators were recategorized into dichotomous variables. An impaired indicator was considered when a score from 1 to 3 (from severe to moderate impairment) was assigned, and not impaired when the indicators had a score of 4 and 5 (low and none). Score 4 (low) was considered not impaired, because the operational magnitudes listed by the instrument do not characterize the indicator as impaired, but as risk.

The variables related to the NO indicators were compared with the clinical variables using Pearson’s Chi-square test and when the expected frequencies were lower than five. Fisher’s test was applied for associative measures of nominal categorical data. To verify the magnitude of the association, the Prevalence Ratio (PR) was used, at a confidence interval (CI) of 95%. In all tests, a statistical significance level of 95% was adopted (p ≤ 0.05).

This study obtained a favorable opinion from the *Comitê de Ética em Pesquisa* - CEP (Research Ethics Committee) of the *Universidade Federal do Rio Grande do Norte*, by means of Advice No. 918.510 and is in accordance with the recommendations of Resolution 466/12 of the National Health Council, which regulates the bioethical principles in research. It is emphasized that prior to data collection, the objectives of the study were explained and the signing of the Informed Consent Form (ICF) was requested by all participants involved in the study, guaranteeing them the right to anonymity and confidentiality of the data obtained.

## Results

Of the 206 patients in the sample of this study, 117 (56.8%) presented the NO Risk of Dry Eyes and 89 (43.2%) clinical signs suggesting Dry Eye. There was a predominance of 108 males (52.4%), with a mean age of 58.41 years (standard deviation of 14.98). Regarding associated comorbidities, systemic arterial hypertension 123 (59.7%) was highlighted, followed by diabetes mellitus 65 (31.6%). In relation to the type of ICU admission, 104 (50.5%) participants were surgical and 102 (49.5%) were clinical. Regarding ventilatory support mechanisms, hemodynamic and neurological, 108 (52.4%) patients used invasive mechanical ventilation therapy, 92 (44.7%) used intravenous vasoactive drugs and 87 (42.2%) used sedatives.

Six indicators were listed to evaluate the degree of ocular impairment of the study patients. Overall, the decrease in tear production 162 (78.6%) and the presence of redness in the conjunctiva 123 (59.7%) were the most prevalent indicators of eyes impairment. The other indicators were more frequent for absence of impairment, namely: incomplete eyelid closure 81.1% (167), excessive tearing 95.1% (196), excessive mucous secretion 78.6% (162) and decreased blinking mechanism 50.5% (104).

Table 1 shows the distribution of the scores of the indicators of the Nursing Result of Dry Eye Severity in relation to the ocular impairment of patients in the ICU.

**Table 1:** Distribution of the indicators of the Nursing Outcome classification (NOC*) Dry Eye Severity by level of severity. Natal, RN, Brazil, 2016

Indicators NOC*	Severe		Substantial		Moderated		Low		None	
	N	%	N	%	N	%	n	%	n	%
Decrease in tear production	48	23.3	59	28.6	35	17	23	11.2	41	19.9
Incomplete eyelid closure	-	-	9	4.4	17	8.3	13	6.3	167	81
Conjunctiva redness	9	4.4	15	7.3	32	15.5	67	32.5	83	40.3
Excessive tearing	5	2.4	4	1.9	1	0.5	-	-	196	95.1
Excessive mucous secretion	2	1	-	-	11	5.3	31	15	162	78.7
Decreased blinking mechanism	85	41.3	7	3.4	6	2.9	4	1.9	104	50.5

* NOC = Nursing Outcomes Classification

For the indicator of tear reduction, the most prevalent scores were severely impaired 48 (23.3%) with Schirmer’s test < 2 mm and substantially impaired 59 (28.6%) with volumetry < 5 mm. In the incomplete eyelid closure indicator, 167 (81%) impairment was not found in the exposure of the ocular surface, followed by a higher frequency for the moderate score (8.3%), with exposure of the lower 1/3 of the cornea.

For the indicator Conjunctiva redness: 5 (40.3%) and 4 (32.5%). The indicator excessive tearing did not show impairment, with higher prevalence of score 5 (95.1%). The most prevalent scores were 5 (78.7%) and 4 (15%). For excessive mucosal secretion, the most prevalent scores were 5 (78.7%) and 4 (15%). In addition, the decreased blinking mechanism was distributed almost equally between scores 5 (50.5%) and 1 (41.3%).

To test the associations between the impairment of clinical indicators with the clinical characteristics and medications used, the bivariate effect of different variables on the NOC (Table 2 and 3) scores was studied. The variables that demonstrated statistical significance with the indicators of the NO under study were presented.

**Table 2:** List of clinical indicators of the Nursing Result Dry Eye Severity of patients admitted to the Intensive Care Unit with hospitalization for neurological disorders, invasive mechanical ventilation and chemosis. Natal, RN, Brazil, 2016

Variables	Impaired	Not impaired	Statistics
Excessive mucous secretion			
Hospitalization for Neurological Disorder			p= 0.048*
Present	3	11	PR = 4.11^†^
Absent	10	182	CI95% = 1.27-13.26^‡^
Invasive Mechanical Ventilation			**p= 0.015^§^**
Present	11	2	PR =5.08^†^
Absent	96	97	CI95% =1.15-22.39^‡^
**Decreased blinking mechanism**			
Hospitalization for Neurological Disorder			**p= 0.016^§^**
Present	11	87	PR = 1.73^†^
Absent	3	105	CI95% = 1.26-2.37^‡^
Invasive Mechanical Ventilation			**p< 0.001^§^**
Present	89	9	PR = 9.15^†^
Absent	18	90	CI95% = 4.87-17.15^‡^
Chemosis			**p< 0.001^§^**
Present	48	50	PR = 2.03^†^
Absent	18	90	CI95% = 1.55-2.65^‡^
**Incomplete eyelid closure**			
Invasive Mechanical Ventilation			**p= 0.001^§^**
Present	23	5	PR = 4.25^†^
Absent	84	94	CI95% = 1.68-10.76^‡^

* Significance test (p-value) related to Fisher’s exact calculation;

† PR = Prevalence Ratio;

‡ CI = Confidence Interval;

§ Significance test (p-value) related to Pearson’s chi-square calculation

**Table 3:** List of clinical indicators of the Nursing Result Dry Eye Severity of patients admitted to an Intensive Care Unit using medications. Natal, RN, Brazil, 2016

Variables	Impaired	Not impaired	Statistics
Incomplete eyelid closure			
Sedatives			p= 0.007*
Yes	19	72	PR= 2.66^†^
No	09	106	CI95% =1.26 – 7.25^‡^
Corticosteroid			p= 0.008*
Yes	16	56	PR = 2.48^†^
No	12	122	CI95% =1.243-4.955^‡^
**Decreased blinking mechanism**			
Sedatives			p< 0.001*
Yes	31	30	PR = 4.63^†^
No	49	96	CI95% =3.11-6.88^‡^
Antibiotic			p< 0.001*
Yes	82	58	PR = 2.41^†^
No	16	50	CI95% =1.54-3.78^‡^
Benzodiazepines			p= 0.001*
Yes	38	19	PR = 1.65^†^
No	60	89	CI95% =1.26-2.16^‡^
Corticosteroid			p< 0.001*
Yes	48	24	PR = 1.78^†^
No	50	84	CI95% =1.35-2.34^‡^
**Conjunctiva redness**			
Benzodiazepines			>p= 0.015*
Yes	24	33	PR = 1.69^†^
No	37	112	CI95% =1.12-2.56^‡^
Vasoconstrictor			p= 0.022*
Yes	31	49	PR = 1.62^†^
No	30	96	CI95% =1.07-2.43^‡^
**Decrease in tear production**			
Antibiotic			p= 0.002*
Yes	93	47	PR = 1.51^†^
No	29	37	CI95% =1.12-2.03^‡^

* Significance test (p-value) related to Pearson’s chi-square calculation;

† PR = Prevalence Ratio;

‡ CI = Confidence Interval

The prevalence ratio demonstrates that the occurrence of impairment of the indicator *Excessive mucosal secretion* is 4.11 higher in the case of hospitalization for neurological disorders and 5.08 higher in the case of Invasive Mechanical Ventilation (IMV), when compared to those who did not have these characteristics.

The occurrence of impairment of the indicator Decreased Blinking Mechanism is 73% higher in the presence of hospitalization for neurological disorders, 9.15 higher in the use of IMV and 2.03 higher in the presence of chemosis, when compared to those who did not have these characteristics.

The impairment of the indicator Incomplete eyelid closure is 4.25 higher when IMV is used.

We also performed the association of the clinical indicators of the Nursing Result Dry Eye Severity of patients admitted to the Intensive Care Unit who was using medications (Table 3).

According to Table 3, the prevalence ratio demonstrates that the occurrence of impairment of the indicator Incomplete eyelid closure is 2.66 higher in the presence of sedation and 2.48 higher when corticosteroids are used, when compared to patients who did not use these medications.

The occurrence of impairment of the indicator Blinking mechanism decreased is 4.63 greater in the use of sedatives, 2.41 greater in the use of antibiotics, 65% greater in the use of benzodiazepines and 78% greater in the use of corticosteroids.

The occurrence of impairment of the indicator Conjunctiva redness is 69% higher in the use of benzodiazepines and 62% higher in patients who use vasoconstrictors. The presence of impairment of the indicator Decrease in lacrimal production is 51% higher when antibiotics are used.

## Discussion

Although Dry Eye is one of the most frequent ophthalmic conditions in the world, studies to evaluate its severity results in Intensive Care Unit need attention^(^ 8 ^)^.

Tears have lubricant and antimicrobial properties that protect the ocular surface against dryness and eliminate particles and microorganisms. When a decrease in lacrimal production occurs, the person becomes vulnerable to external agents and prone to develop the dry eye^(^ 13 ^)^.

It is possible to measure this decrease from the Schirmer test, which is considered normal when the values are above 10mm/5min. A study carried out in ICU of a hospital in Turkey showed that the decrease in lacrimal production quantified by the Schirmer test of less than 10 mm/5 min was found in 70% of the sample and a score of less than 5 mm/5 min in 40% of patients^(^ 13 ^)^. In this study, we observed that ICU patients obtained the indicator severe Decrease in lacrimal production and moderately impaired (< 2mm and < 5 mm, respectively), since the frequencies were higher for scores 1 (23.3%) and 2 (27.2%).

The eyelid closure involves an active process of contraction of the orbicular muscle and inhibition of the upper eyelid lifting muscle. Incomplete eyelid closure is one of the main indicators with potential to trigger eye disorders, due to total or partial exposure of the eye surface and consequent excessive tear evaporation^(^ 14 ^)^. In this study, if evaluated individually, it was found that the incomplete eyelid closure was absent (score 5) in most of the sample (81.1%). However, patients using invasive mechanical ventilation, sedatives and corticosteroids presented a higher occurrence for the impairment of this indicator. However, when associated with invasive mechanical ventilation, with the use of sedatives and corticosteroids, it showed a remarkable statistical significance.

This result related with the findings of other authors demonstrate that the occurrence of Dry Eye is closely associated with the degree of lagophthalmos and depth of sedation. A recent study revealed that on the first day of ICU stay, 86.3% of patients had total eyelid closure in the right eye and 85.3% in the left eye, however, after one week of using sedatives, 20.3% evolved with incomplete eyelid closure in the right eye and 24.3% in the left (increased involvement in 6.6% and 9.6%, respectively)^(^ 14 ^)^.

The use of IMV, according to the literature, may impair ocular homeostasis. There is a discussion about the use of this ventilatory device to cause an increase in intraocular pressure resulting in altered perfusion. In addition, it triggers venous stasis that promotes fluid retention and consequent conjunctival edema (chemosis). All this succession of events related to IMV promotes the incomplete eyelid closure^(^ 15 ^-^ 16 ^)^.

Patients using sedatives have the inability to completely close the eyelids by relaxing the oculomotor muscles. This inability leads to exposure of the conjunctiva and results in drying of the ocular surface. The degree of exposure is linked to the depth of sedation^(^ 17 ^)^. Other studies have also found associations between dry eye signs and corticosteroids, but plausible explanations for this association was not found^(^ 3 ^,^ 8 ^)^.

The *conjunctiva redness* results from the presence of dilated blood vessels on the ocular surface and is mainly triggered by insufficient lubrication of the eyes or their exposure to air with low humidity. In order to evaluate the problems involving the Dry Eye in ICU patients, a study identified that conjunctival hyperemia was the most frequently observed ocular problem, present in 56.25% of the patients analyzed^(^ 13 ^)^.

Similarly, the results of this study showed that the score 4 (conjunctival hyperemia) was the second most prevalent (32.5%) in detriment of the other indicators and, when statistically associated, it is possible to observe an association with the use of vasoconstrictors and benzodiazepines.

Medications such as vasoconstrictors that inhibit parasympathetic activity are strongly related to eye dryness because of reduce lacrimal production. Benzodiazepines are related as a predisposing factor to hyperemia, although not directly. Acting as sedatives and promoting muscle relaxation by decelerating organic function, thus, incomplete eyelid closure promotes corneal exposure, evaporation of the tear film, dryness and consequent hyperemia, which indicates a process of acute inflammation^(^ 3 ^)^.

Regarding the indicator Excessive mucous secretion, it is discussed that the hyperactivity of the caliciform glands in the conjunctiva may lead to an excessive production of mucous secretion and is usually the result of a previous inflammatory state. When present, the secretion is located on the ocular surface and its aspect is of whitish coloration in format of filaments or plates. A recent study found that the absence of ocular hyperemia (p<0.001), of mucous secretion (p<0.045) and the non-use of sedatives (p<0.025) were significantly associated with the absence of the Dry eye^(^ 3 ^)^.

In this study, this indicator was statistically associated with the use of invasive mechanical ventilation. It was discussed above that the use of IMV significantly alters the homeostasis of the eye by predisposing the appearance of conjunctival edema and successively the dry eye. Mucous secretion occurs in the last phase of this process of dryness caused by exposure as an inflammatory sign of the mucin-producing glands, which is a late sign of the phenomenon^(^ 16 ^)^.

The indicator Decreased blinking mechanism showed statistical significance with: hospitalization for neurological disorders, IMV, chemosis, sedatives, antibiotics, benzodiazepines and corticosteroids.

The blinking reflex periodically promotes uniform distribution of the tear over the entire eye surface. When it is diminished, the tear film ruptures occurs and the dryness protection mechanism fails. Spontaneous and effective blinking of the eyes (5 to 10 blinks/second) prevents evaporation of the tear between blinks, eliminates foreign bodies, excludes visual stimuli and ensures the optical quality of the eye by spreading and distributing the tear film over the eye surface^(^ 18 ^)^.

The IMV and chemosis variables converge to the decrease in the blinking mechanism, as discussed above, the use of this ventilatory device promotes the accumulation of liquids in the conjunctiva that originates the chemosis^(^ 3 ^)^.

Regarding sedatives, the variable with a higher prevalence ratio, studies point out that, as in lagophthalmos, the loss of the blinking reflex is observed in patients with low level of consciousness and high depth of sedation level. Sedatives and muscle relaxants may suppress the act of blinking, preventing an adequate distribution of the tear on the ocular surface^(^ 19 ^)^.

The indicator Excessive tearing was present in only 10 patients and there was no significant association with any of the variables. For further investigation of this indicator in the ICU environment, a study is necessary to identify the different phases of the Dry Eye, because excessive tearing is considered an indicator for the initial phase and the low frequency of this may be associated with the characteristics of the sample.

Among the limiting factors of the study, its design (transversal) stands out, which does not allow the continuous monitoring of the sample. Thus, it is emphasized the importance of developing longitudinal studies with these NOC scales, given their relevance for a more assertive targeting of the impairment of the problems found.

## Conclusion

This study allowed assessing the degree of ocular impairment based on the clinical indicators of the Nursing Result Dry Eye Severity in accordance with the objective defined.

The clinical indicators of the nursing outcome Dry Eye Severity most severely impaired were the decrease in lacrimal production and the decreased blinking mechanism. Hospitalization for neurological disorders, use of invasive mechanical ventilation, presence of chemosis, use of sedatives, vasoconstrictors, antibiotics, benzodiazepines and corticosteroids were associated with the presence of some indicators of Dry Eye Severity.

Finally, we emphasize the importance of care practices aimed at the prevention of diseases, performed not only by nursing professionals, but also by all those involved in health care, in accordance with the National Program for Patient Safety.

## References

[1] Craig JP, Nichols KK, Caffery B, Dua HS, Joo CK, Liu Z (2017). TFOS DEWS II Definition and Classification Report. Ocul Surf.

[2] Martinez JD, Galor A, Betancourt NR, Cervantes AL, Beltrán F, Zárate JO (2016). Frequency and risk factors associated with dry eye in patients attending a tertiary care ophthalmology center in Mexico City. Clin Ophthalmol.

[3] Araújo DD, Almeida NG, Silva PMA, Ribeiro NS, Werli-Alvarenga A, Chianca TCM (2016). Prediction of risk and incidence of dry eye in critical patients. Rev. Latino-Am. Enfermagem.

[4] Holland EJ, Whitley WO, Sall K, Lane SS, Raychaudhuri A, Zhang SY (2016). Lifitegrast clinical efficacy for treatment of signs and symptoms of dry eye disease across three randomized controlled trials. Curr Med Res Opin.

[5] Herdman TH, Kamitsuru S (2018). NANDA International Nursing Diagnoses: Definitions and Classification, 2018-2020.

[6] Bulechek GM, Butcher HK, Dochterman JM, Wagner CM (2018). Nursing Interventions Classification (NIC).

[7] Moorhead S, Johnson M, Maas M, Swanson E (2018). Nursing Outcome Classification (NOC).

[8] Fernandes APNL (2015). Dry Eye Severity in patients admitted to the Intensive Care Unit: Concept analysis and build settings.

[9] Mantovani VM, Acelas ALR, Lucena AF, Almeida MA, Heldt EPS, Boaz SK (2017). Nursing Outcomes for the Evaluation of Patients During Smoking Cessation. Int J Nurs Knowl.

[10] Oliveira AR, De Araujo TL, De Carvalho EC, Costa AG, Cavalcante TF, Lopes MV (2015). Construction and validation of indicators and respective definitions for the nursing outcome Swallowing Status. Rev. Latino-Am. Enfermagem.

[11] Malta M, Cardoso LO, Bastos FI, Magnanini MMF, Silva CMFP (2010). STROBE initiative: guidelines on reporting observational studies. Rev Saúde Pública.

[12] Câmara VGN, Araújo JNM, Fernandes APNL, Botarelli FR, Silva AB, Costa RA, Silva HP, Pitombeira DO, Santos MMP, Ferreira MA, Vitor AF (2016). Methods for detection of dry eye in critically ill patients: An Integrative Review. Int Arch Med.

[13] Saritas TB, Bozkurt B, Simsek B, Cakmak Z, Ozdemir M, Yosunkaya A (2013). Ocular surface disorders in intensive care unit patients. Sci Wld J.

[14] Kuruvilla S, Peter J, David S, Premkumar PS, Ramakrishna K, Thomas L (2015). Incidence and risk factor evaluation of exposure keratopathy in critically ill patients: A cohort study. J Crit Care Med.

[15] Jammal H, Khader Y, Shihadeh W, Ababneh L, Aljizawi G, AlQasem A (2012). Exposure keratopathy in sedated and ventilated patients. J Crit Care.

[16] Alansari MA, Hijazi MH, Maghrabi KA (2015). Making a Difference in Eye Care of the Critically Ill Patients. J Intensive Care Med.

[17] Kousha O, Kousha Z, Paddle J (2018). Exposure keratopathy: Incidence, risk factors and impact of protocolised care on exposure keratopathy in critically ill adults. J Crit Care.

[18] França CFSM, Fernandes APNL, Carvalho DSRP, Xavier SSM, Ferreira MA, Vitor AF (2016). Evidence of interventions for the risk of dry eye in critically ill patients: An integrative review. App Nurs Res.

[19] Horng CT, Chou HL, Tsai KL, Hsiao HY, Lin SY, Huang SF (2014). The observation for ocular surfasse diseares in respiratory care center in regional teaching hospital in southem Taiwan. Life Sci J.

